# *Aspergillus fumigatus* phosphoethanolamine transferase gene *gpi7* is required for proper transportation of the cell wall GPI-anchored proteins and polarized growth

**DOI:** 10.1038/s41598-019-42344-1

**Published:** 2019-04-10

**Authors:** Haomiao Ouyang, Ting Du, Hui Zhou, Iain B. H. Wilson, Jinghua Yang, Jean-Paul Latgé, Cheng Jin

**Affiliations:** 1 State Key Laboratory of Mycology, Institute of Microbiology, Chinese Academy of Sciences, Beijing, 100101 China; 2 Department of Chemistry, University of Natural Resources and Life Sciences, Vienna, A-1190 Austria; 3 Guangxi Academy of Sciences, Nanning, 530007 Guangxi China

## Abstract

In fungi many proteins, which play important roles in maintaining the function of the cell wall and participating in pathogenic processes, are anchored to the cell surface by a glycosylphosphatidylinositol (GPI) anchor. It has been known that modification and removal of phosphoethanolamine (EtN-P) on the second mannose residue in GPI anchors is important for maturation and sorting of GPI anchored proteins in yeast and mammalian cells, but is a step absent from some protist parasites. In *Aspergillus fumigatus*, an opportunistic fungal pathogen causing invasive aspergillosis in humans, GPI-anchored proteins are known to be involved in cell wall synthesis and virulence. In this report the gene encoding *A. fumigatus* EtN-P transferase GPI7 was investigated. By deletion of the *gpi7* gene, we evaluated the effects of EtN-P modification on the morphogenesis of *A. fumigatus* and localization of GPI proteins. Our results showed that deletion of the *gpi7* gene led to reduced cell membrane GPI anchored proteins, the mis-localization of the cell wall GPI anchored protein Mp1, abnormal polarity, and autophagy in *A. fumigatus*. Our results suggest that addition of EtN-P of the second mannose on the GPI anchor is essential for transportation and localization of the cell wall GPI-anchored proteins.

## Introduction

Species belonging to *Aspergillus*, *Candida*, *Cryptococcus*, and *Pneumocystis* are major fungal pathogens causing invasive infections among immunocompromised patients. About 1.5–2 million people are killed by fungal infection every year, which is more than those killed by malaria or tuberculosis^1,2^. Even with drug treatments, the mortality associated with fungal infections is still high, therefore there is a urgent need to develop new drugs. However, our limited knowledge of fungal pathogens is the major barrier for the development of techniques of early diagnosis and novel therapies^1^.

Among 185 recognized *Aspergillus* species, about 20 species can cause infections in humans. *Aspergillus fumigatus* is the second clinically important fungal pathogen, accounts for about 65% of all invasive infections in humans and is the mostly encountered species in pulmonary infections^3^. Mortality associated with *A. fumigatus* infection exceeds 50% despite treatment with antifungal agents. The reason why *A. fumigatus* is a major fungal pathogen is not understood yet. It has been shown that *A. fumigatus* is able to adapt to high temperatures, oxidative stress, nutrient limitations, and hypoxic conditions, as well as to synthesize secondary metabolites and secrete enzymes for nutrient uptake^4–7^. Recently, it has been shown that efficient internalization of conidia by epithelial cells, delayed germination, and hyphal growth parallel to the epithelium might be the causes for the success of *A. fumigatus*^8^. Although all these abilities can contribute to the survival of *A. fumigatus* inside the human body, no single characteristic determines the pathogenicity of *A. fumigatus*. On the other hand, the fungal cell wall has been thought as an ideal target for drug development since it is essential for growth and morphogenesis of fungi, yet is absent from human cells^5^. Therefore, understanding the biosynthesis of the *A. fumigatus* cell wall is of utmost importance.

Although the cell wall of *A. fumigatus* is a rigid structure composed of mannoproteins, glucan, and chitin, it must be remodeled during its polarized growth, including germination, hyphal growth, cell separation, and conidiation. Glycosylphosphatidylinositol (GPI)-anchored proteins are known as enzymes able to modify cell wall polymers and indispensable for the continuous shape adaptation of the cell wall, and proteins involved in mating, filamentation or adhesion^9^. In addition, certain GPI-anchored proteins are known as the major components of mannoproteins in the cell wall, which are important for cell wall biogenesis and cell wall assembly^10^. It has been shown that suppression of the GPI anchor biosynthesis leads to a defect in cell wall and increased cell death in *A. fumigatus*^11^. Indeed, similarly to trypanosomatids^12^, it has been recently confirmed that the GPI anchor is a promising antifungal target^13^.

The biosynthesis of GPI anchor in yeast and mammalian cells has been well characterized^10,14,15^. The biosynthesis of GPI anchor is initiated with sequential modification of phosphatidylinositol (PI) by the addition of sugars and ethanolaminephosphate (EtN-P), thus forming a complete precursor lipid in the ER. Genes required for the attachment of the EtN-P have been identified in yeast and human cell: *PIG-N*/*MCD4* are responsible for the transfer of EtN-P onto the first mannose residue of GPI anchor, *GPI7* transfers EtN-P onto the second mannose residue, and *PIG-O*/*GPI13* transfer EtN-P onto the third mannose residue. Also *PIG-F* is possibly invovlved in the addition of EtN-P, as *PIG-F* mutants fail to transfer EtN-P to the third mannose residue in mammalian cells^16^, while the yeast homologue (*GPI11*) is rather required for the addition of EtN-P to the second mannose^17^. On the other hand, in *T. cruzi*, *T. brucei* and *P. falciparum*, only the third mannose is modified by EtNP and only *GPI13* homologues are found^18^.

In yeast, deletion of the *MCD4*, *GPI11*, or *GPI13* gene is lethal, while deletion of the *GPI7* only causes a defect in cell wall integrity, such as an increased chitin content and a decreased protein content^17,19–23^. In the *gpi7*-null mutant, the expressions of cell wall GPI-anchored proteins are severely affected. On the other hand, the expressions of membrane GPI-anchored proteins, such as Gas1p, are not affected^23,24^. Gpi7p is also essential for separation of the daughter cells after cytokinesis. Egt2p, the GPI-anchored protein that is normally concentrated in the septum, was mislocalized in the *gpi7* mutant^25^. In *Candida albicans*, *GPI7* is involved in chlamydospore formation, budding patterns, and cell shape of *C. albicans*^26,27^. The *C. albicans GPI7* mutant cells are more sensitive to the lytic action of macrophages, indicating that a functional GPI anchor is required for hyphal formation of *C. albicans* and perturbation of the GPI anchor synthesis results in hypersensitivity to host defense^23^. In the dimorphic yeast *Yarrowia lipolytica GPI7* is involved in invasive growth^28^. These findings clearly demonstrate that the addition and removal of the EtN-P on the second mannose of the GPI glycan are important process that ensures the transportation of the proteins essential for cell wall structure and remodeling, especially during polarized hyphal growth of yeast.

Although the GPI biosynthetic pathways of *A. fumigatus* and yeast are comparable^29^, in contrast to the yeast *GPI7*s, the biological significance of the *A. fumigatus gpi7* has not yet been clarified. In this study, the significance of EtN-P transferase Gpi7 was evaluated by deletion of the *gpi7* gene in *A. fumigatus*. We demonstrated that deletion of the *gpi7* in *A. fumigatus* led to abnormal polarity, altered levels of plasma membrane GPI-anchored proteins (GPI-PMP), mislocalization of cell wall GPI anchor proteins (GPI-CWP), and autophagy.

## Results

### Construction of the Δ*gpi7* mutant

**Figure 1:**
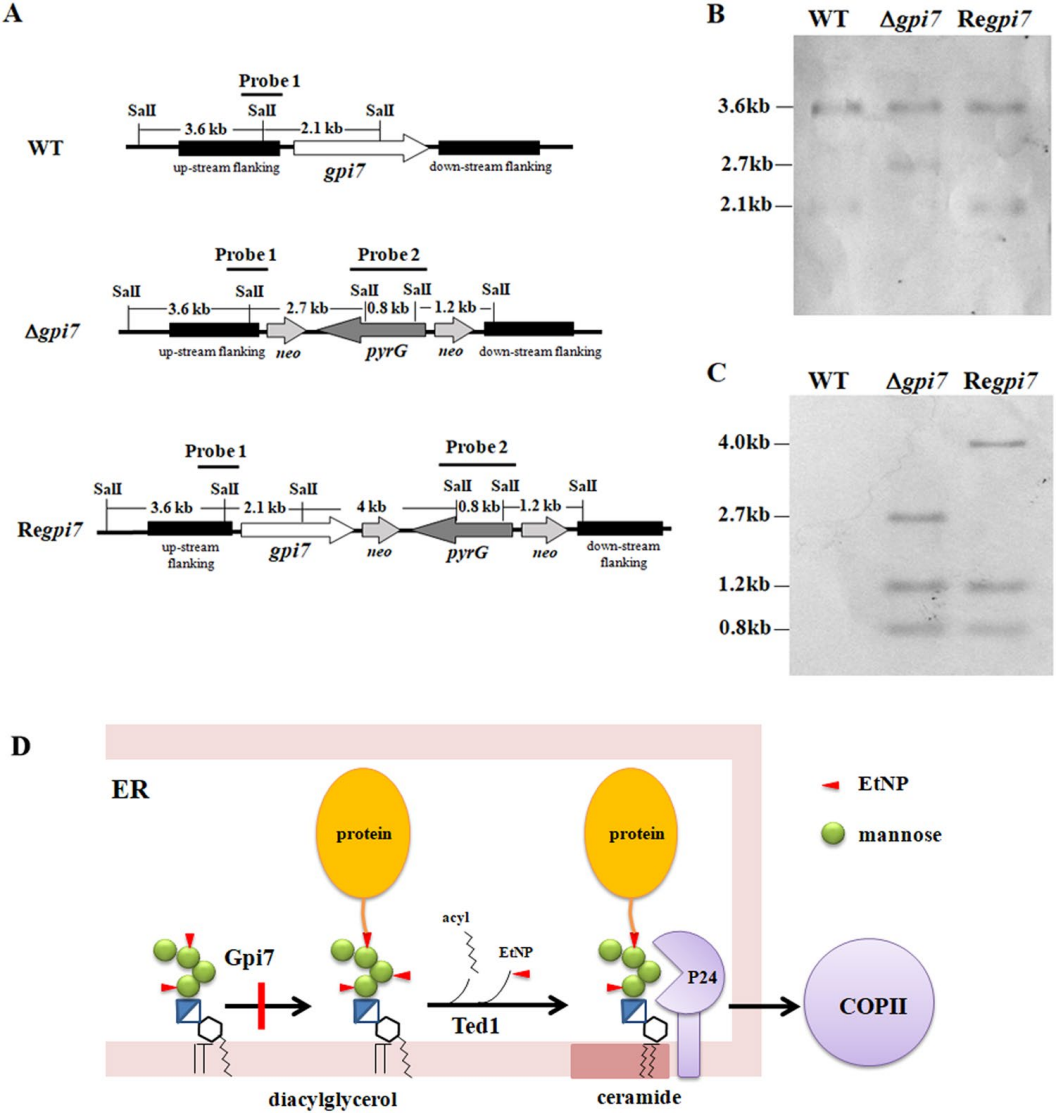
Confirmation of the mutant and revertant by Southern blotting. The null mutant Δ*gpi7* was constructed by replacing of the *gpi7* gene with *pyrG* and the revertant strain Re*gpi7* was constructed by introducing of the *gpi7* gene into the mutant as described under Methods. Both wild-type (WT) and revertant strains were confirmed by Southern blotting. Probe 1 and probe 2, amplified from the up-stream flanking sequence of the *gpi7* and the *pyrG* gene respectively, were used as probes. Genomic DNA digested with Sal I was probed with Probe 1 (**B**) or Probe 2 (**C**) as described under Methods. In the ER, the second mannose of GPI-anchor is modified by Gpi7 with EtN-P and then protein will be attached to GPI anchor. Once GPI anchored protein is formed, the EtN-P on the second mannose will be removed by Ted1p/PGAP5. Then p24 protein, a cargo receptor in COPII vesicle, can recognize the mature GPI-anchored protein by direct binding to the second mannose, which leads to the formation of a specialized COPII vesicle^43^ (**D**).

By searching the database of *A. fumigatus* Af293 genome with the *S. cerevisiae* Gpi7p at the NCBI website (http://blast.ncbi.nlm.nih.gov/Blast.cgi), the candidate *gpi7* gene (AFUA_6G05260) was retrieved, which encodes a protein that shares the similarity of 44.8% and the identity of 30.5% to the yeast Gpi7p. To evaluate the significance of the *gpi7* gene in *A. fumigatus*, the null mutant was constructed by replacing the *gpi7* gene with *pyrG*. Also, a revertant strain was constructed. Both mutant and revertant strain were confirmed by Southern blotting. As shown in Fig. 1B, when probe 1 was used to detect SalI-digested genomic DNA, a 3.6-kb and a 2.1-kb fragment were detected in the wild-type (WT) and revertant (Re*gpi7*), while a 3.6-kb and a 2.7-kb fragment were detected in the mutant Δ*gpi7*. When probe 2 was used to detect SalI-digested genomic DNA, 0.8-kb, 1.2-kb and 4.0-kb fragments were detected in the revertant, while 0.8-kb, 1.2-kb, 2.7-kb fragments were detected in the mutant Δ*gpi7* (Fig. 1C). These results clearly demonstrated that the *gpi7* gene was replaced by *pyrG* gene in the Δ*gpi7* mutant and the *gpi7* was re-introduced in the revertant strain.

### Phenotype analysis

**Figure 2:**
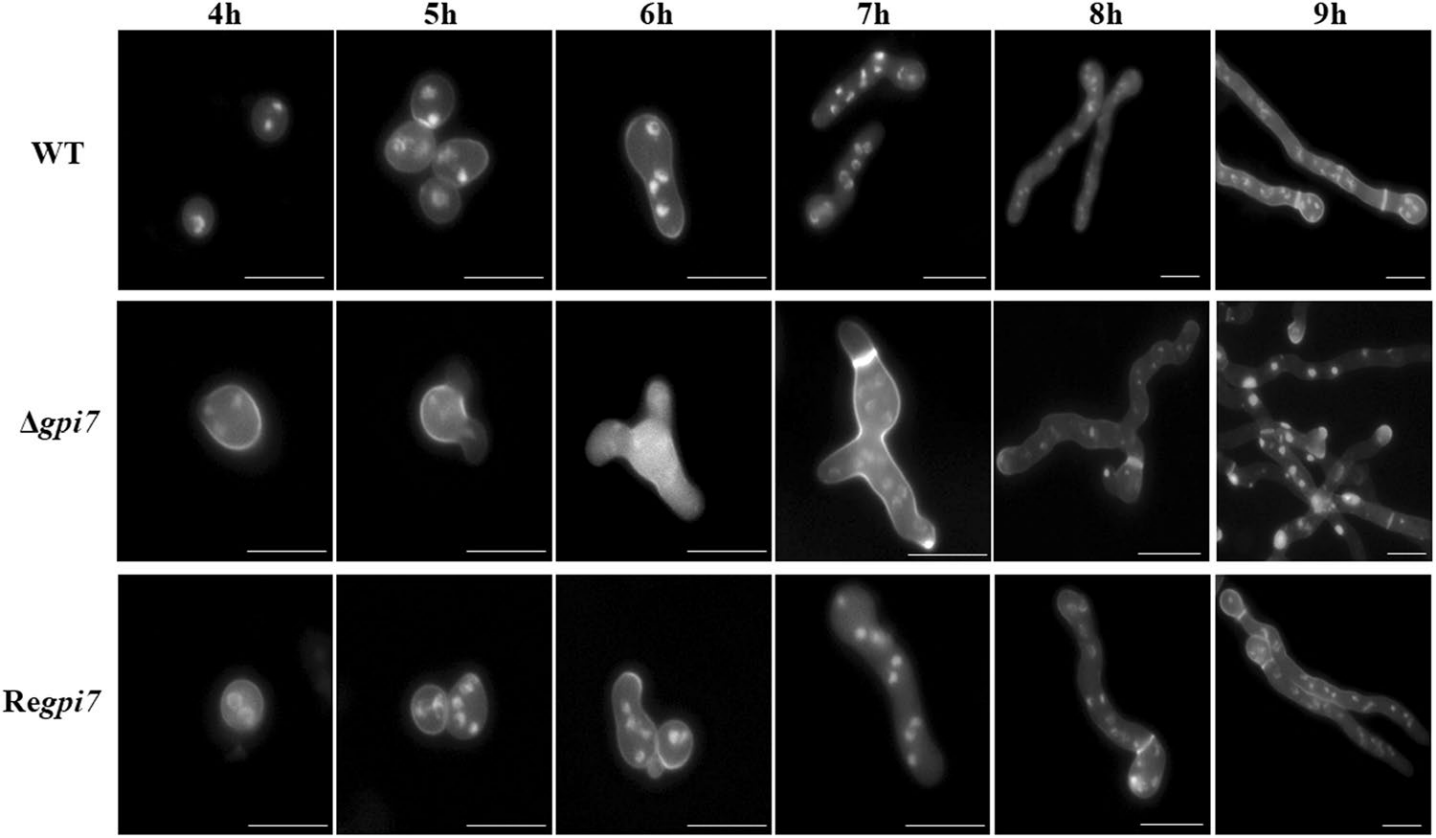
Conidia germination of the Δ*gpi7* mutant. Observation of conidia germination in microscopy. Spores of the WT, Δ*gpi7* and Re*gpi7* were stained with DAPI and calcofluor white. Scale bar is 10 µm.

As life cycle of filamentous fungi starts from spore germination, continues with hyphal growth and ends by sporulation, we carefully analyzed the phenotypes of the mutant at these stages. As can be seen in Fig. 2 and Table 1, the mutant spores germinated earlier than the WT and revertant. About 55% of the mutant spores established their polarity at 4 hours. After incubation for 5 hours, 16% of the mutant spores generated the second germling tube and 98% of the second germling tube formed 120° angle with the first germling tube. The second germling tube of the mutant occurred after the second mitosis, and 50% of the mutant spores showed multiple budding sites at 6 hours. After 7 h of incubation, the mutant strain experienced four rounds of mitosis and 32% of them formed the first septa, while in the WT the first septa was formed after 8 hours. After 9 hours, 12% of the mutant showed third germling tube and 93.1% of the mutant exhibited swollen hyphal tips with strong staining by calcofluor white, suggesting a hyper branching and an accumulation of chitin at the hyphal tips. These results demonstrated an abnormal polarized growth during germination stage of the mutant.

**Table 1:** Statistics of germination of the Δ*gpi7* mutant.

Time (h)	WT number of germ tube (%)				Δ*gpi7* number of germ tube (%)				Re*gpi7* number of germ tube (%)			
	0	1	2	≥ 3	0	1	2	≥ 3	0	1	2	≥ 3
4	95 ± 2	5 ± 1	0	0	45 ± 2	55 ± 2	0	0	92 ± 3	7 ± 2	0	0
5	61 ± 3	35 ± 2	1 ± 0	0	20 ± 1	62 ± 3	16 ± 2	2 ± 0	55 ± 4	43 ± 1	2 ± 0	0
6	45 ± 1	54 ± 2	1 ± 0	0	4 ± 1	42 ± 2	44 ± 3	6 ± 1	22 ± 2	65 ± 3	4 ± 1	0
7	7 ± 1	88 ± 4	5 ± 1	0	3 ± 0	24 ± 1	66 ± 2	7 ± 1	11 ± 3	80 ± 5	6 ± 2	2 ± 1
8	4 ± 0	91 ± 5	6 ± 2	1 ± 0	2 ± 1	15 ± 1	71 ± 2	12 ± 2	2 ± 0	89 ± 4	8 ± 1	2 ± 1

10 mL of liquid CM was inoculated with 10^6^ spores in a petri plates containing sterilized glass coverslips and incubated at 37 °C. The coverslips with adhering germinated conidia were taken out, fixed in PFA solution (3.7% paraformaldehyde, 50 mM phosphate buffer, pH 7.0, and 0.1% Triton X-100), observed and counted under differential interference contrast microscope. 100 cells were counted and repeated 3 times. Results are presented as mean ± SD.

**Figure 3:**
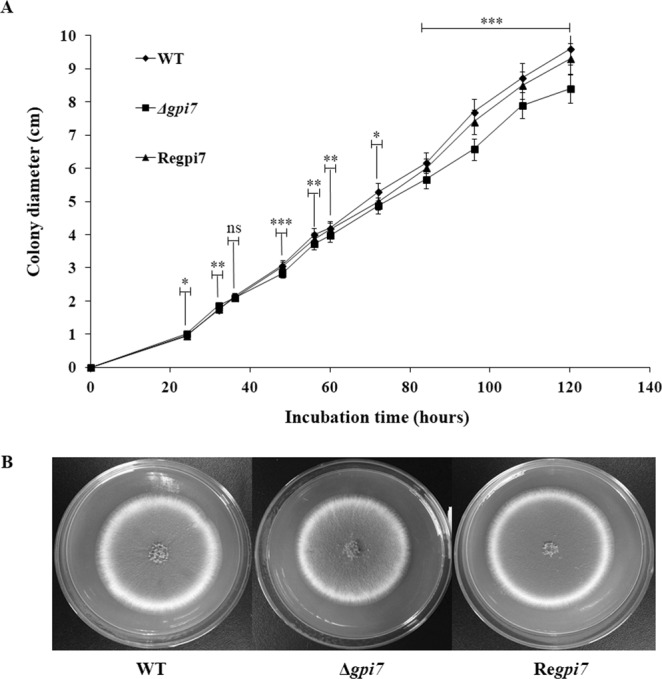
Hyphal growth of the Δ*gpi7* mutant. 10^4^ conidia of the wild-type (WT), Δ*gpi7* mutant, or Re*gpi7* revertant strain were spotted on CM plate respectively and incubated at 37 °C. At specific time points, the diameter of colony was measured and averaged. The mean diameter was used to plot against the growth kinetics (**A**). The strains were incubated at 37 °C for 120 h (**B**). This experiment was carried out triplicate. The statistical analysis was performed based on the diameter of clony between Δ*gpi7* and WT. Mean and SD are presented; ns, not significant; *p < 0.05; **p < 0.01; ***p < 0.001.

**Figure 4:**
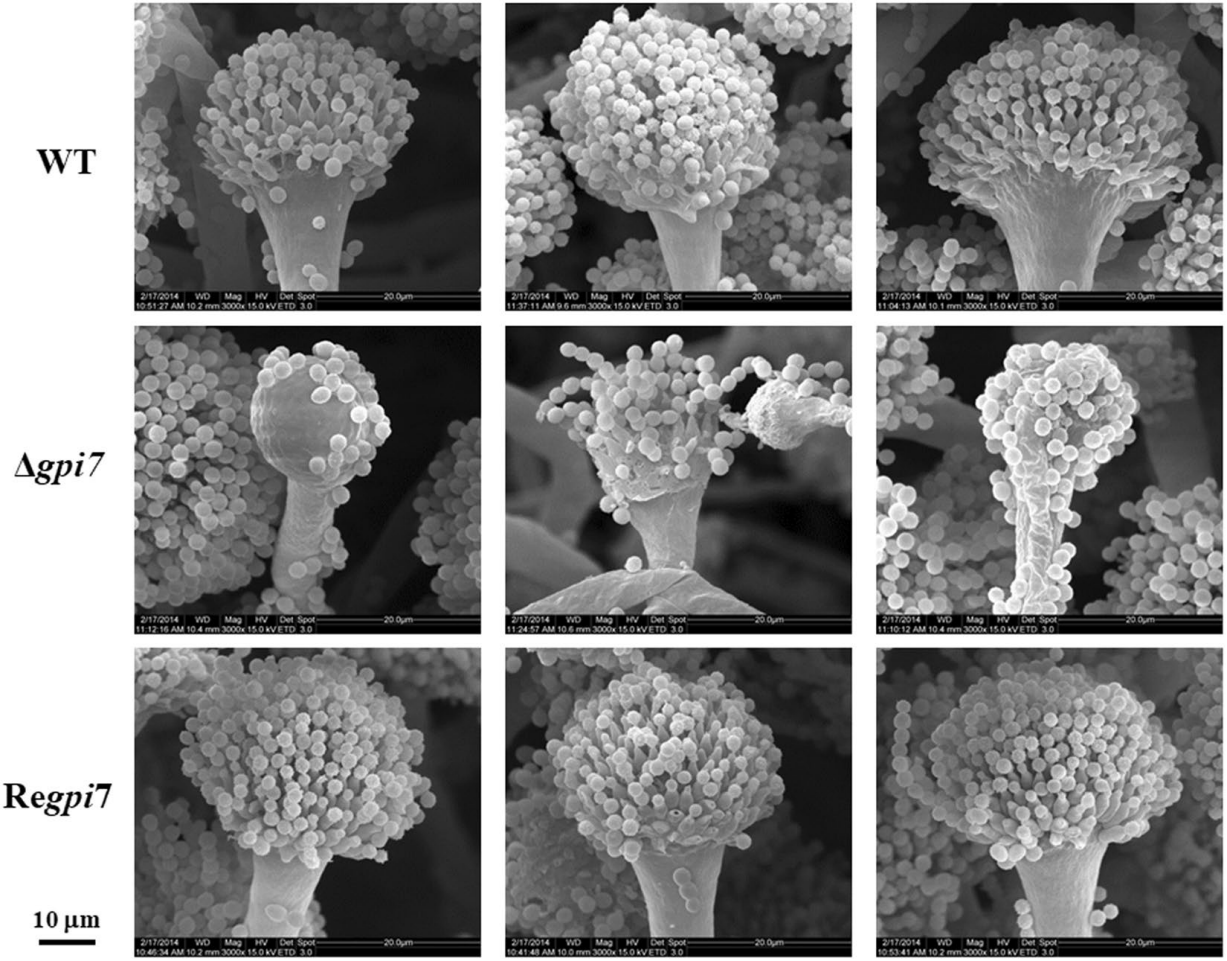
Phenotype of the Δ*gpi7* mutant. The mycelia cultivated in liquid CM at 37 °C were collected, fixed and examined using an Environmental Scanning Electron Microscope. Scale bar is 10 µm.

**Figure 5:**
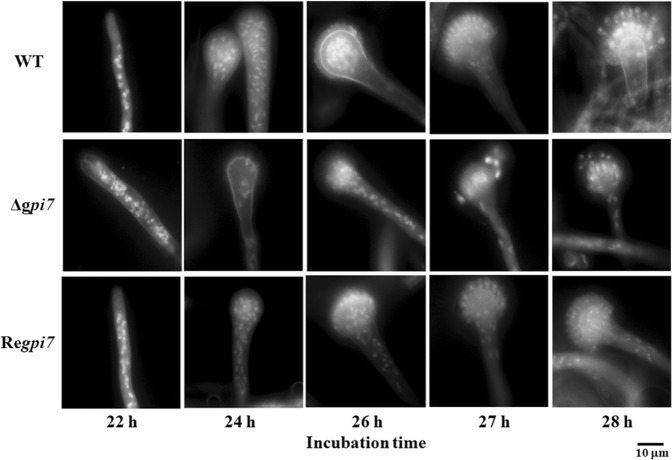
Sporulation of the Δ*gpi7* mutant. 2 × 10^8^ spores were inoculated into 200 ml liquid CM and incubated at 37 °C at 200 rpm, then stained with 10 μg/ml Calcofluor white and 1 mg/ml 4′-6-diamidino-2-phenylindole (DAPI) as previously described^36^, and detected by fluorescence microscopy. Scale bar is 10 µm.

At hyphal growth stage, the growth rate of the mutant was slightly decreased after 36 h of incubation on solid complete medium (CM) at 37 °C (Fig. 3). Under scanning electron microscopy (SEM), the mutant apparently lost its ability to form phialide as compared with the WT (Fig. 4), which was further confirmed by staining of the mutant with calcofluor white and DAPI (Fig. 5). Spore counting revealed that conidiospores produced by the mutant were only 30% of the WT (Table 2). These observations indicated that deletion of the *gpi7* resulted in an abnormal polarized growth during sporulation stage.

**Table 2:** Conidiospores produced by the mutant.

Strain	0 h	72 h***
WT	1 × 10^7^	(7.3 ± 0.4) × 10^9^
Δ*gpi7*	1 × 10^7^	(2.7 ± 0.2) × 10^9^
*Regpi*7	1 × 10^7^	(6.4 ± 0.4) × 10^9^

The statistical analysis of spores production. The number of start spores in WT, Δ*gpi7* and Re*gpi7* is 10^7^ and newly produced spores are counted after 72 hours. The statistical analysis was performed based on the average numbers of newly produced conidiospores after 72 hours. Results are presented as mean ± SD. ***p < 0.001.

**Figure 6:**
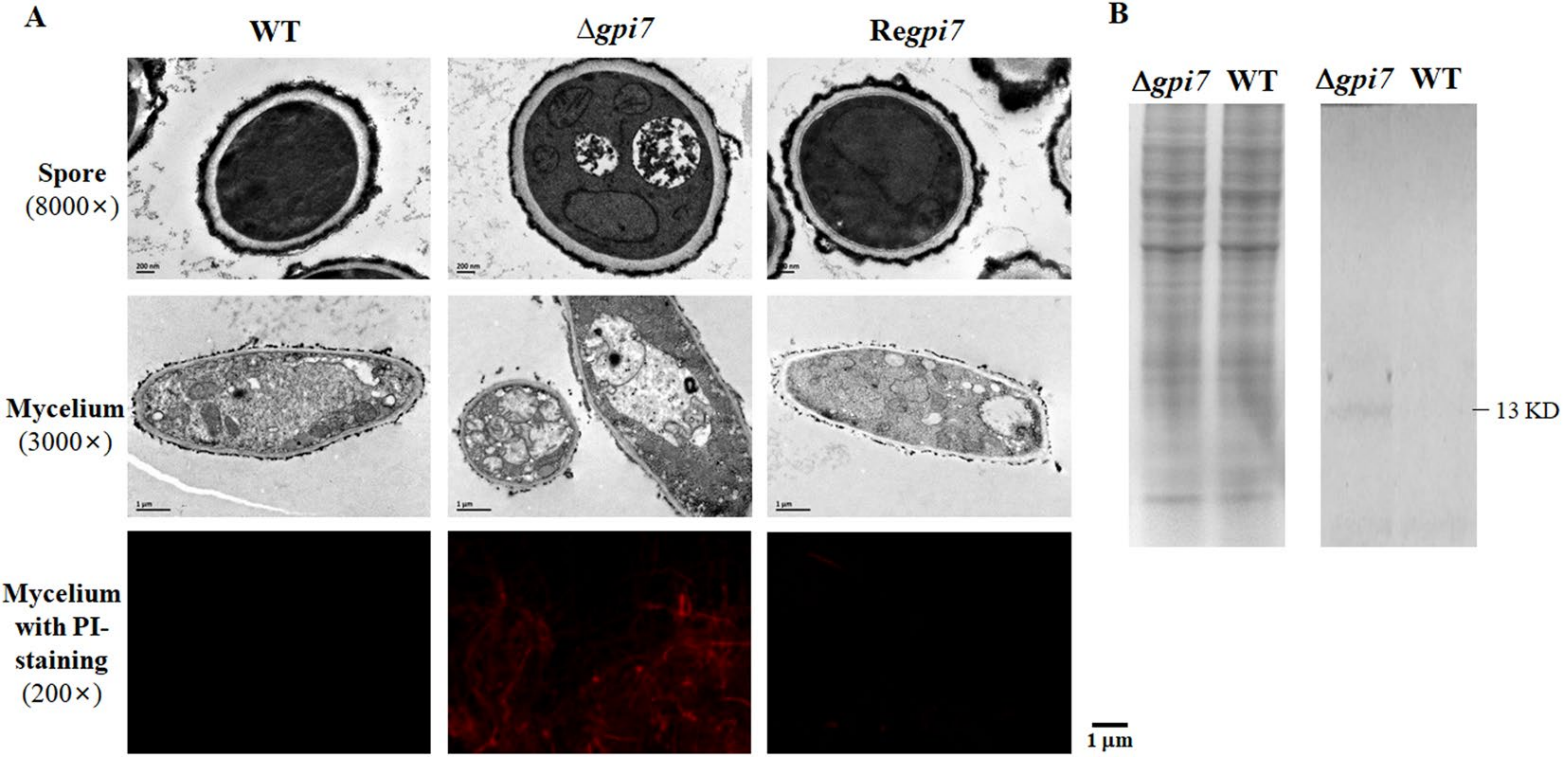
Autophagy induced in the Δ*gpi7* mutant. In (**A**), the conidia of the WT and mutant grown on solid medium were fixed and examined by electron microscopy as described in Methods; in (**B**), intracellular proteins extracted from the WT and mutant (left panel) were separated on SDS-PAGE and transferred onto PVDF membranes (Millipore). The membrane was blocked with 5% fat-free milk in TBST for 2 h at room temperature, and then incubated with using anti-LC3II antibody at 4 °C overnight. Then the membrane was washed three times with TBST buffer and incubated with an AP-conjugated secondary antibody for 1 h at room temperature. After washing three times with TBST buffer, bands were detected with NBT/BCIP reagent. Scale bar is 1 µm.

The mutant did not show increased sensitivity to calcofluor white, Congo red, DTT, tunicamycin, or Nikkomycin (data not shown), suggesting that the cell wall integrity was not affected and ER-stress was not induced in the mutant. We further examined the cell wall under transmission electron microscopy (TEM). As shown in Fig. 6A (upper and middle panel), the mutant did not show any significant change on its cell surface as compared with the WT; however, enlarged vacuoles were found in 45% of the mutant spores and hyphae, which is similar to autophagy induced by suppression of GPI anchor synthesis^11^. Propidium iodide (PI) staining revealed that about 47.8% of the mutant mycelia at logarithmic phase were positive stained (Fig. 6A, lower panel), while only around 20.4% of the WT mycelia were positive stained. Autophagy mediates the turnover of long-lived proteins and excess or aberrant organelles, however, under certain situations severe autophagy also lead to programmed cell death. Autophagy involves in the formation of double membrane-bound structure called autophagosome, which engulfs material to be degraded in lytic compartment^30^. During formation of autophagosome, Atg8/LC3 (lipidation) conjugation system directly contributes to the elongation of isolation membranes and the maturation of autophagosome^31^. Therefore, Atg8/LC3 is used as reliable markers for the induction and progression of autophagy. By using LC3II antibody a 13-kD band was detected in the Δ*gpi7* mutant, while no such band was detected in the WT (Fig. 6B). These results suggested an activation of autophagy in the mutant cells.

### Localization of GPI anchored proteins

To evaluate the impact of the *gpi7* on synthesis of GPI-anchored proteins, we determined the GPI anchored proteins in the mutant. As summarized in Table 3, GPI-anchored cell wall proteins (GPI-CWPs) in the mutant were decreased by 29.8%, while GPI-anchored plasma membrane proteins (GPI-PMPs) were increased by 18%, and the extracellular proteins also decreased by 26.9% in the mutant.

**Table 3:** Determination of proteins in the *∆gpi7* mutant.

	Dry weight (mg)^ns^	GPI-CWP (mg)**	GPI-PMP (mg)***	Extracellular protein (mg)***	Intracellular protein (mg)^ns^
WT	710 ± 35	8.0 ± 0.4	26.1 ± 0.1	16.1 ± 0.3	29.6 ± 0.1
Δ*gpi7*	760 ± 28	5.6 ± 0.3	30.8 ± 0.1	11.8 ± 0.3	29.2 ± 0.1

10^8^ spores were cultivated in CM medium at 37 °C, 200 rpm, for 36 hours. GPI-CWP, cell wall GPI anchored proteins; GPI-PMP, cell membrane GPI anchored proteins. The statistical analysis was performed between WT and Δ*gpi7*. Results are presented as mean ± SD. ns, not significant; *p < 0.05; **p < 0.01; ***p < 0.001.

Real time RT-PCR analysis revealed that 6 genes encoding GPI anchored protein were up-regulated or down-regulated in the Δ*gpi7* mutant (Table 4). Gel1 (AFUA_2G01170), a β-1,3-glucanosyltransferase responsible for the elongation of cell wall β-1,3-glucan side chain^32^, is the homolog of *S. cerevisiae* Gas1p or *C. albicans* Phr1p and Phr2p. Gel1 is a GPI-PMP^32,33^; Mp1 (AFUA_4G03240), a cell wall galactomannoprotein^34^, is a strict GPI-CWP covalently linked to the cell wall; Ecm33 (AFUA_4G06820) is a GPI-PMP^33,35^. In the mutant the RNA levels of the *mp1, gel1* and *ecm33* were increased by 13.2-, 6.4-, and 3.4-fold, respectively. All three genes encode glycoproteins. Gel1p and Ecm33p are modified by both N- and O-linked glycans, whereas Mp1 is modified by O-glycans only. Moreover, Mp1p is identified as a protein covalently linked to the cell wall β-glucans via its O-glycans^35^.

**Table 4:** Expression of the genes encoding GPI anchored protein.

Locus tag	Protein name	Annotation	Expression (fold)
AFUA_4G06820	Ecm33	GPI anchored protein	3.4
AFUA_2G01170	Gel1	GPI anchored protein	6.4
AFUA_3G13200	Gel7	GPI anchored protein	2.8
AFUA_4G03240	Mp1	GPI anchored protein	13.2
AFUA_5G01970	GPDA	control	1

The real-time PCRs were performed on a separate batch of RNA. The presented expression fold is in relation to control WT.

**Figure 7:**
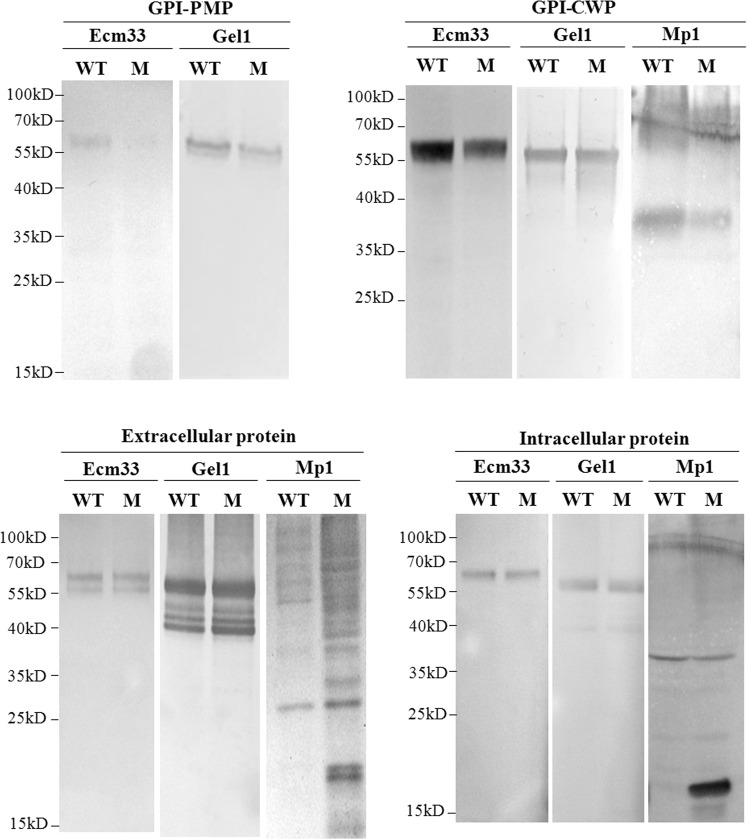
Localization of GPI anchored proteins. Cell wall GPI proteins (GPI-CWP) were released from the cell wall by hydrofluoride-pyridine (HF); Cell membrane proteins (GPI-PMP) were released from membrane fraction by HF, membrane fraction was prepared by ultracentrifuge. Extracellular proteins were extracted from culture supernatant by precipitation and intracellular proteins were obtained from the cell lysate^35^. Western blotting was carried out with anti-Gel1, anti-Ecm33, and anti-Mp1 antibody, respectively. Lower levels or degradation of Gel1 (452 amino acids with 2 N-glycosylation sites and some O-glycosylation sites, 56 kDa), Ecm33 (398 amino acids with 9 N-glycosylation sites and some O-glycosylation sites, 61 kDa) and Mp1 (284 amino acids with some potential O-glycosylation sites, 42 kDa) can be observed in certain cellular subfractions.

Using anti-Gel1, anti-Mp1 and anti-Ecm33 antibodies^35,36^, the distribution of GPI anchored proteins was detected in the mutant. No significant change was observed with intracellular soluble form of Ecm33 or Gel1 proteins; however, membrane-bound Gel1 and Ecm33 were obviously decreased in the mutant (Fig. 7). As for GPI-CWP, Mp1 was decreased in the cell wall of the mutant. In addition, a dramatic degradation of both intracellular and extracellular soluble form of Mp1 to 18 kDa was observed (Fig. 7), which is consistent with its significantly increased expression level of the *mp1* gene (Table 4). These results indicated that the transport and localization of Ecm33, Gel1, and Mp1 were affected in the mutant, especially cell wall GPI protein Mp1.

**Figure 8:**
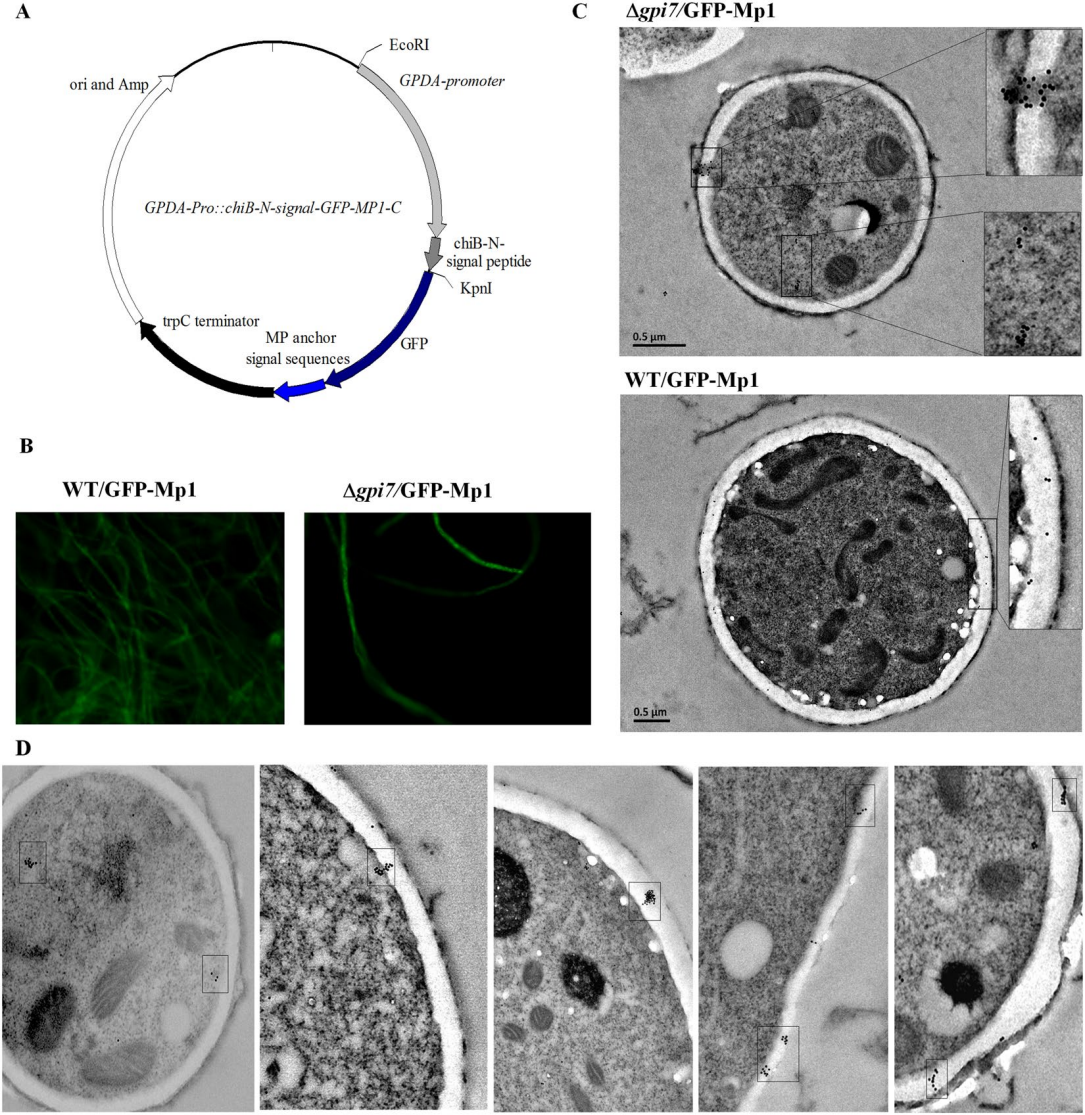
Distribution of the GFP-Mp1 chimeric protein in the Δ*gpi7* mutant. In (**A**), plasmid GPDA-Pro::chiB-N-signal-GFP-Mp1-C; in (**B**), expression of chimeric GFP-Mp1 in the WT and Δ*gpi7* mutant; and in (**C**), GFP-Mp1 was detected with Anti-GFP polyclonal antibody (Clontech) by using immuno gold labeling method as described under Material and Methods; in (**D**), GFP-Mp1 in the Δ*gpi7* mutant was detected with anti-GFP polyclonal antibody. Gold particles are marked with boxes.

Previously, we have shown that GFP-Mp1, a chimeric protein with an *Af*ChiB1 N-terminal signal peptide sequence fused to the N terminus and a GPI signal sequences of Mp1 fused to C terminus of GFP, is localized in the cell wall^35^. To track the intracellular transport and localization of Mp1 protein, the vector pGFP/Mp1 harboring the gene encoding GFP-Mp1 (Fig. 8A) was introduced into the WT and mutant strain, respectively. The transformants were screened for GFP expression under fluorescence microscopy in the mutant and WT (Fig. 8B). Gold immuno-labeling with anti-GFP antibody was carried out to track the transport and localization of GFP-Mp1 in both the WT and mutant. As shown in Fig. 8C, in the WT GFP-Mp1 proteins were evenly distributed in the cell wall, while in the mutant GFP-Mp1 proteins were aggregated inside the cell and crossed the cell wall in a vesicle-like manner (Fig. 8C,D), suggesting that GFP-Mp1 in the mutant was directly secreted, instead of evenly transported to the cell wall.

## Discussion

In most eukaryotes including yeast and mammals, the biosynthesis of GPI anchor is initiated with sequential modification of PI by the addition of sugars and EtN-P in the ER^10,14–16^. Once the complete anchor is formed, the GPI transamidase complex recognizes the GPI signal at the carboxyl-terminal of substrate proteins, cleaves the GPI signal from the proprotein substrate, and covalently links the new carboxyl terminus to a GPI moiety. Thereafter, both the lipid and glycan moieties of the GPI anchor are remodeled in the ER, which are two essential steps for the transportation of GPI-anchored proteins via COPII vesicles^37–39^. The first step of the GPI anchor remodeling involves removal of acyl chain from the inositol group by Bst1/PGAP1^40^. The second step of remodeling is removal of EtN-P from the second mannose of the GPI glycan by PGAP5 and replacement of the acyl chains^37,41,42^. In yeast, clustering of GPI-anchored proteins at ER exit sites requires remodeling of the GPI acyl chains, perhaps inducing association of the cargos with lipid microdomains^39^.

Recent investigations suggest that p24 proteins monitor the status of GPI-anchor maturation in both yeast and mammalian cells^38,39^. Manzano-Lopez *et al*. found that p24 protein, a cargo receptor in COPII vesicle, recognizes the mature GPI-anchored protein by direct binding to the second mannose on which EtN-P has been removed by Ted1p, the yeast PGAP5 ortholog. Binding of the mature GPI-anchored protein to the p24 complex induces recruitment of the COPII subunit Lst1 to ER exit sites and formation of a specialized COPII vesicle^43^. Therefore, it is proposed that Gpi7, Ted1, and p24 constitute a quality control system in the ER to prevent packaging of immature and misfolded GPI-anchored proteins into COPII vesicles (Fig. 1D).

In yeast, deletion of *GPI7* only affects cell wall integrity. The expressions of cell wall GPI-anchored proteins are severely affected, whereas the expressions of membrane GPI-anchored proteins, such as Gas1p, are not affected^17,19–24^. In *C. albicans GPI7* is involved in chlamydospore formation, budding patterns, cell shape, and hyphal formation^23,27^. These findings clearly demonstrate that the addition of the EtN-P onto the second mannose of the GPI anchor is important for cell wall proteins and polarized hyphal growth of yeasts.

Similar to *C. albicans*, in this study we showed that the *A. fumigatus gpi7* was involved in polarized growth during spore germination, hyphal growth, and conidiation. On the other hand, but in contrast to yeast, despite the amount of GPI-CWPs was decreased in the mutant (Table 3), deletion of the *gpi7* did not affect the cell wall integrity of *A. fumigatus*. Considering that Gpi7 is one of the proteins that consist quality control system of GPI-anchored proteins^38,39^, it was reasonable to assume that deletion of the *gpi7* would cause accumulation of GPI-anchored proteins in the ER of the mutant. However, to our surprise, the mutant did not show any increased sensitivity to DTT or tunicamycin. Interestingly, we observed enlarged vacuole and autophagosome in the mutant cells. Indeed 45% of the mutant spores and hyphae were found to contain autophagosome or enlarged vacuole. Meanwhile the mutant mycelia at logarithmic phase showed increased death. These observations suggested an induction of autophagy in the mutant, which was further confirmed by positive detection of LC3 (microtubule associated protein 1 light chain 3, LC3) in the mutant. Therefore, it can be concluded that deletion of the *gpi7* led to autophagy instead of ER-stress in *A. fumigatus*.

Previously we have shown that suppression of the complete GPI anchor synthesis pathway via deletion of the *PIG-A/GPI3 N*-acetylglucosaminyltransferase gene leads to ER-stress, autophagy and necroptosis in *A. fumigatus*^11^. It appears that interfering with the addition of the EtN-P to the second mannose residue of GPI anchor neither completely blocked the transfer of proteins to immature GPI anchor nor resulted in the similar phenotypes as suppression of the GPI *N*-acetylglucosaminyltransferase. As matter of fact, some GPI anchored proteins were still synthesized and transported to cell membrane in the *gpi7* mutant (Fig. 7), which suggests a leakage of the quality control system of GPI-anchored proteins and the mutant could compensate for the low efficiency of the transfer of proteins to immature GPI anchor by upregulating the expression of proteins, such as Gel1, Gel7, Ecm33, and PhoA, by 3.4–6.4-fold (Table 4). Although this upregulation did not completely restore the amount of membrane-bound Gel1 or Ecm33, it was enough to compensate the cell wall integrity in the mutant.

On the other hand, although the expression of the *mp1* gene was elevated to 13.2-fold of the WT, the amount of Mp1 in the cell wall was significantly decreased, while intracellular and extracellular soluble Mp1 protein levels were increased and degraded dramatically. These results suggest that most of Mp1 protein molecules in the ER of the mutant could not be transferred to an immature GPI anchor; as accumulation of Mp1 in the ER did not induce ER-stress, it is likely that accumulated proteins that were unable to attach to immature GPI anchor were degraded by an autophagy-related mechanism.

Previously, we have shown that Mp1 is a GPI-CWP, which is covalently attached to cell wall β-glucan cell via its O-glycan. The GPI signal sequence of Mp1 can direct the localization of chimeric protein GFP-Mp1 in the cell wall^35^. Therefore, in this study we tracked the transportation and localization of GPI-CWP by expressing GFP-Mp1 in both WT and mutant. Our results showed that GFP-Mp1 was evenly distributed in the cell wall of the WT, while GFP-Mp1 molecules were aggregated in a small area with a diameter less than 0.2 µm inside the cell or crossing the cell wall of the mutant. As the cell size of *A. fumigatus* is quite small as compared with mammalian cell, we were unable to track the intracellular transport of GFP-Mp1 by confocal analysis. Somehow, based on the autophagosome and vesicle-like aggregation of GFP-Mp1 in the mutant, it is reasonable to conclude that the GPI anchoring of GPI-CWPs were greatly affected in the mutant and unanchored GPI-CWPs were degraded by an autophagic pathway and secreted via exosomes.

In summary, in this study we found that deletion of the *gpi7* gene led to abnormal polarized growth, reduced conidiation, and increased autophagy in *A. fumigatus*. Although lack of EtN-P on the second mannose residue of the GPI anchor did not affect the localization of GPI-PMPs that are required for cell wall synthesis, addition of EtN-P to the second mannose residue is essential for transport and localization of GPI-CWPs, thereby suggesting that the transport of the latter is more dependent on the quality control system for GPI-anchor proteins.

## Methods

### Strains and growth conditions

*Aspergillus fumigatus* strain YJ-407 (China General Microbiological Culture Collection Center, CGMCC0386) was maintained on potato glucose (2%) agar slant^44^. *A. fumigatus* strain CEA17 which is a *pyrG* mutant strain^45^, a kind gift from C. d’Enfert, Institute Pasteur, France. The bacterial strain used for transformation and amplification of recombinant DNA was *E. coli* DH5α. *A. fumigatus* strain YJ-407 was grown at 37 °C on complete medium (CM), or minimal medium (MM) with 0.5 mM sodium glutamate as a nitrogen source^45^, solidified with 1.5% (w/v) agar when required. Uridine and uracil were added at a concentration of 5 mM for strain CEA17. Mycelia were harvested from strains grown in complete liquid medium at 37 °C with shaking at 200 rpm. Conidia for spore innoculation were prepared by growing *A. fumigatus* strains on solid complete medium with uridine and uracil (CMU) for 48 hours at 37 °C, harvested with 0.1% Tween 20 and washed twice with distilled water. Its concentration was confirmed by haemocytometer counting and flat dilution counting.

### Construction of the ∆*gpi7* mutant and revertant strain

Flanking regions of the *gpi7* gene were amplified from *A. fumigatus* strain YJ-407 genomic DNA. The upstream and downstream flanking regions of the *gpi7* gene were digested with KpnI*/*EcoRI and EcoRI/XbaI, respectively. By insertion of the flanking regions into the corresponding sites of pBluescript II SK (+) (Stratagene), p5′3′SK carrying the flanking regions of the *gpi7* gene was obtained. The *pyrG*-blaster cassette (8.6 kb) released by digestion of pCDA14 (kindly provided by C. d’Enfert, Institut Pasteur, France)^46^ with HpaI was cloned into the site between the up- and down-stream non-coding regions of the *gpi7* to yield pGPI7-pyrG, which was linearized at a unique NotI site and transformed into the CEA17 strain by protoplast transformation^47^. 0.55 mg/mL 5-fluorotic acid (5-FOA) was used to obtain *∆gpi7*^-^*pyrG*^-^ from *∆gpi7*.

The revertant strain was constructed by the replacement of the *pyrG* in the *∆gpi7* mutant with the up- stream non-coding regions and *gpi7* and *pyrG* and down-stream non-coding regions of the *gpi7*. The *gpi7* with its up-stream 4.6-kb and down-stream 1.6-kb non-coding regions was amplified by PCR, cloned into the corresponding sites of pBluescript II SK(+), and former Pgpi75′3′SK. The *pyrG* (3.5 kb) with BamHI restriction sites was cloned from pCDA14 by PCR (primer pair: *RepyrG-N* and *RepyrG-C*). Then, the *pyrG* segment digested by BamHI site, was inserted into Pgpi75′3′SK between *gpi7* and down-stream non-coding regions of the *gpi7*. Primers are listed in Table 5. The transformants were chosen first by PCR and then confirmed by Southern blot analysis using the up-stream non-coding region as a probe (Fig. 1), which was labeled following the protocol of DIG labeled hybridization kit (Roche Applied science Cat. NO. 1093657).

**Table 5:** Primers used in this study.

Primer	Sequence (5′-3′)
Del-gpi7-up-5′	GGGGTACCGCGGCCGTTGTCCCTGGTTTGCTCG
Del-gpi7-up-3′	CTGTTGCTGTAGGGATGGTG
Del-gpi7-down-5′	GGAATTCCACGTGAAAATGTCTGACCCAAGCAGC
Del-gpi7-down-3′	GCTCTAGAACTTCTTGTCCCGAGTACAGC
Rev-pyrG-5′	CGGGATCCCAGTAACTGAAGTGACATAGGGTTCG
Rev-pyrG-3′	CGGGATCCCTAATACCGCCTAGTCATAGCAG
Rev-gpi7-up-5′	GAGTGTTCGAGCCAGTGTAACCAAG
Rev-gpi7-up-3′	CGGGATCCCTCGTTTAACGACAAGCAACTGAATG
Rev-gpi7-down-5′	CGGGATCCAAAATGTCTGACCCTAAGCAGC
Rev-gpi7-down-C	CGAGCTCGCGGCCGCACTTCTTGTCCCGAGTACAGC
pyrg-5′	TAAGCCGCTGGTCAATGTTATCTGG
pyrg-3′	ATGCCATTCTCCCCATGAAGTCTG
Afgpi7–5′	CGTAAGCGCATAAGTCATAAC
Afgpi7-3′	GAGAGATCGTATATGTCC
Ecm33-5′	GGCTTTCCTCAAATACGCTCTCCC
Ecm33-3′	ATCGCTCTGGCTGGAAATCGTG
Gel1-5′	AGGGCAATGCCTTCTTCAAGGG
Gel1-3′	TTGAACTTGGCAATGTCACGCTTG
Gel7-5′	CTGTTCGGACAATTTCAGCTGTTGG
Gel7-3′	GCATTGTTCCGTGTTGATGAGTGG
Mp1-5′	GAAGCTTGTCAGCACCATCAACTCC
Mp1-3′	TCTTGGAGATGAGGTCGTCGATGAC
GpdA-5′	AGTAATCGCAGGTCTTCCGTAACGC
GpdA-3′	GATGAAGGGGTCGTTGACAGCAAC

### Phenotype analysis of the mutant

Growth of the *A. fumigatus* strains was carried out onto solid CM or MM at 37 °C. For morphological observation, 2 × 10^8^ spores were inoculated into 200 ml liquid CM and incubated at 37 °C at 200 rpm, then stained with 10 μg/ml Calcofluor white (Sigma) and 1 mg/ml 4′-6-diamidino-2-phenylindole (DAPI; Sigma) as previously described^36^, prior to fluorescence microscopy using a Zeiss Imager A2 (Zeiss, Japan). For propidium iodide (PI) staining, 2 × 10^8^ conidia were inoculated in 200 ml of CM and incubated at 37 °C with 200 rpm. The mycelia were collected, stained with PI, and then examined under the fluorescence microscope.

For scanning electron microscopy (SEM) or transmission electron microscopy (TEM), the mycelia cultivated in liquid CM at 37 °C were collected and fixed as previously described^11,36^ prior to examining the sections with a Tecnai Spirit (120 kV) transmission electron microscope (FEI, USA). For chemical analysis of the cell wall, cell wall components were isolated and determined as previously described^11^.

### Real-time PCR

Total RNAs were extracted using TRIZOL (Invitrogen, Carlsbad, CA, USA). The cDNA synthesis was performed with 5 μg RNA using RevertAid^TM^ First Strand cDNA Synthesis Kit (Fermentas, USA). The PCR reaction was done with SYBR^®^ (Takara, DRR041A) as previously described^11^. Samples isolated from different strains and different times were tested in triplicate. The primers used for confirmation of the differentially expressed genes are listed in Table 5.

### Proteins and western blotting

1 × 10^8^ conidia were inoculated into 200 ml CM at 37 °C for 36 h with 200 rpm. Proteins extraction and western blotting was treated as previously described^35^.

### Immunogold staining and electron microscopy

1 × 10^8^ conidia were inoculated into 200 ml CM at 37 °C for 36 h with 200 rpm. *A. fumigatus* mycelia were harvested, Immunogold staining was performed as previously described^35^. Samples were examined using a Tecnai Spirit (120 kV) transmission electron microscope (FEI Company, Eindhoven, Netherlands). Negative controls were performed by reacting samples with an irrelevant murine IgG or with the immune-conjugates alone.

### Statistical analysis

The Student t test was used in statistical analysis of experimental data. The P value less than 0.05 was considered as statistically significant.
